# A comparison between the effect of trans-anethole and metformin on biochemical parameters of polycystic ovary syndrome in rats

**DOI:** 10.22038/AJP.2021.55679.2785

**Published:** 2021

**Authors:** Faezeh Moradi Negahdari, Mousa-Al-Reza Hadjzadeh, Zahra Gholamnezhad, Zahra Samadi Noshahr, Zakieh Keshavarzi

**Affiliations:** 1 *Applied Biomedical Research Center, Mashhad University of Medical Sciences, Mashhad, Iran*; 2 *Division of Neurocognitive Sciences, Psychiatry and Behavioral Sciences Research Center, Mashhad University of Medical Sciences, Mashhad, Iran*; 3 *Department of Physiology, Faculty of Medicine, Mashhad University of Medical Sciences, Mashhad, Iran*; 4 *Department of physiology, North Khorasan University of Medical Sciences, Bojnurd, Iran*

**Keywords:** PCOS, Testosterone, Trans-anethole, Metformin, Insulin, Dehydroepiandrosterone

## Abstract

**Objective:**

The effect of trans-anethole and metformin on biochemical and hormonal changes of testosterone-induced Polycystic ovary syndrome (PCOS) in rats was investigated.

**Materials and Methods:**

Female Wister rats (n=48) were randomly divided into six groups: control; PCOS; PCOS+metformin (300 mg/kg); and PCOS+trans-anethole (20, 40, and 80 mg/kg). PCOS was induced by intraperitoneal injection of testosterone (1 mg/kg/day) for 35 days. After induction of PCOS, trans-anethole and metformin were given orally for 30 days. Finally, blood sugar, insulin, lipid profile, and testosterone and dehydroepiandrosterone (DHEAS) as well as animals’ weight, and water and food intake were determined.

**Results:**

In all treated and untreated PCOS groups, serum testosterone levels were significantly increased compared to the control group (p<0.001 for all groups). Treatment of rats with trans-anethole or metformin significantly reduced serum levels of cholesterol, insulin, triglycerides, testosterone and DHEAS (only in PCOS+trans-anethole groups) compared to the PCOS group (p<0.01-p<0.001). Weight gain in the PCOS animals increased significantly compared to the control group (p<0.001), while in the metformin- and trans-anethole (40 and 80)-treated animals it decreased significantly compared to the PCOS group (p<0.01-p<0.001).

**Conclusion:**

These results showed that trans-anethole significantly decreased serum levels of insulin, DHEAS and blood lipids. It can be concluded that trans-anethole ameliorates PCOS biochemical and hormonal change in PCOS rats; therefore, it might be suggested as a beneficial remedy for further clinical evaluations in PCOS patients.

## Introduction

Polycystic ovary syndrome (PCOS), with its clinical and biochemical features, is prevalent in approximately 6 to 8% of woman during the reproductive years (Soares Júnior et al., 2015 ▶). Hyperandrogenism is a major feature of PCOS, and it occurs due to the overproduction of androgens in the ovaries and high levels of insulin which stimulate ovarian androgen production and suppress liver’s production of sex hormone-binding globulin (Cassar et al., 2016 ▶). In PCOS, there is an increase in serum levels of testosterone, DHEAS, and prolactin. Numerous metabolic abnormalities such as hypertension, visceral obesity, hyperinsulinemia, dyslipidemia and manifestation of male symptoms (hair growth and muscle type), weight gain, infertility and amenorrhea occur in these patients (Maqbool et al., 2019 ▶). The metabolic disturbances seen in PCOS directly increase the risk of type 2 diabetes (DMT2), coronary heart disease (CHD), cardiovascular disease (CVD), and endometrial cancer (Franks, 1995 ▶). 

Different types of anti-PCOS drugs are prescribed for these patients, such as clomiphene citrate, metformin, letrozole, and spironolactone (Lowenstein, 2006 ▶). However, so far, no definitive and specific treatment has been proposed for PCOS treatment. In most of PCOS patients, these drugs have undesirable side effects. Therefore, several medicinal plants with high anti-PCOS activities have been proposed for treatment of PCOS (Zehra and Khursheed, 2018 ▶). These herbal remedies may slow down the development of complications in PCOS patients with minimal or no side effects. One of these herbs that are widely used in Iran is *Foeniculum vulgare* (fennel). The plant seed contains protein, fat, sugar and mucilage, calcium, phosphorus, iron, potassium and vitamins A (Shams Ardekani et al., 2005 ▶). *F*. *vulgare* has antimicrobial and antioxidant properties due to the presence of flavonoids, terpenoids, carotenoids and coumarins, and it is used in the treatment of many diseases (Singh et al., 2006 ▶). Numerous studies have indicated the role of *F*. *vulgare* in many gynecological diseases, including premenstrual disorders (PMS), heavy menstrual bleeding, painful menstruation, menopause, vaginal atrophy, and sexual dysfunction in postmenopausal women (Mahboubi, 2019 ▶). Moreover, previous studies showed that fennel extract was able to increase serum estrogen, progesterone and prolactin levels in female mice, but decrease the levels of testosterone, insulin and luteinizing hormone (LH) (Malini and George, 2018 ▶; Zehra and Khursheed, 2018 ▶). 

Trans-anethole is one of the most important active ingredients of fennel seed. Therapeutic properties of trans-anethole include antioxidant, anti-hirsutism, anti-diabetic, anti-inflammatory and estrogenic effects (Rahimi and Ardekani, 2013 ▶). However, the therapeutic properties of trans-anethole in PCOS rats have not been determined. 

Metformin is one of the first-line drugs for the treatment of DMT2, and it is widely prescribed to treat the symptoms and complications of PCOS (Xia et al., 2018 ▶). Metformin has shown to improve insulin resistance and menstrual cycle, decrease serum androgen levels, induce ovulation and increase the rate of pregnancy in PCOS patients (Di Pietro et al., 2015 ▶).

Therefore, in the present study, the effects of trans-anethole and metformin on biochemical and hormonal changes in a rat model of testosterone-induced PCOS were investigated.

## Materials and Methods

**Drugs**

Drugs and materials were obtained as follows: testosterone from Caspian Company (Iran), trans-anethole and metformin from Sigma Company (India), and olive oil from Farabi Company. Kits used for determination of triglycerides (TG) and total cholesterol (TC), were purchased from Pars Azmoon Company (Iran, Tehran). Enzyme immunoassay kits were used for measurement of insulin (Cayman Chemical, USA), and testosterone and DHEAS (New England Nuclear, Boston, MA) levels.

**Animals**

Forty-eight female Wister rats (180-210 g body weight) were used in this study. The animals were purchased from the Animal House of Mashhad University of medical School, and kept under standard conditions (22±2°C with 12 hr-12 hr light-dark cycles) and they had free access to food and water, during the experiment. The study was carried out in accordance with ethical principles and policies approved by the Committee on Animal Research of Mashhad University of Medical Sciences (Ethical No. IR.MUMS.MEDICAL.REC.1399.075).

**Experimental design**

Animals were randomly divided into six groups as: control; PCOS; PCOS treated with metformin (300 mg/kg) (P+met); PCOS treated with 3 doses of trans-anethole (20, 40, and 80 mg/kg respectively presented as Trans20, Trans40 and Trans80). To induce PCOS, olive oil-diluted testosterone (1 mg/kg/day) was injected intraperitoneally for 35 days (Beloosesky et al., 2004 ▶). After induction of PCOS, treatments were given orally by gavage for 30 days. During the treatment period (last month of the study) weight was measured weekly but water and food intake was measured every 3 days until the end of the experiment. At the end of the study, the rats were fasted overnight, and under deep anesthesia with urethane (1.6 g/ kg), blood was collected from the retro orbital sinus of the animals and then, rats were sacrificed. Blood was centrifuged at 1500 rpm for 15 min and serums were collected and stored at -20°C for measuring the levels of sex hormones (testosterone and DHEAS), insulin, and serum lipids.

**Statistical analysis**

Normality of data distribution was checked by the method of Kolmogorov and Smirnov in Instat. Data are expressed as mean±SEM and were analyzed by one-way analysis of variance (ANOVA) followed by Turkeys' *post-hoc* test. p values <0.05 were considered significant. Graphs were drawn using Microsoft Excel. 

## Results

**Effect of metformin and trans-anethole on serum testosterone level**

**Figure 1:**
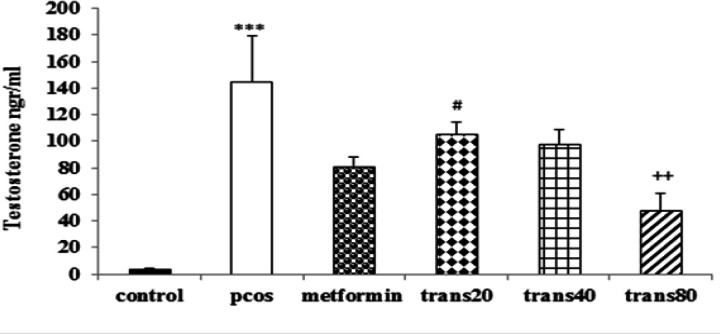
Effect of metformin and trans-anethole on testosterone levels in control, PCOS, PCOS+metformin (P+met), and PCOS+trans-anethole (Trans 20, 40, and 80) groups. Values are expressed as mean±SEM. ***p<0.001, compared to the control group; ^++^p<0.001, compared to the PCOS group; #p<0.05, comparison between Trans 20 and Trans 80 group

There was a significant increase in serum testosterone level of the PCOS group compared to the control group (p<0.001). The group treated with Trans80 showed a significant decrease in testosterone level compared to the PCOS group (p<0.001). There were no significant differences between testosterone levels of the P+met group and trans-anethole-treated groups. However, the testosterone level of Trans80 group was significantly lower than the Trans20 group (p<0.05), but it was not was not significantly different from the control group (Figure 1). 

**Effect of metformin and trans-anethole on serum DHEAS level**

**Figure 2:**
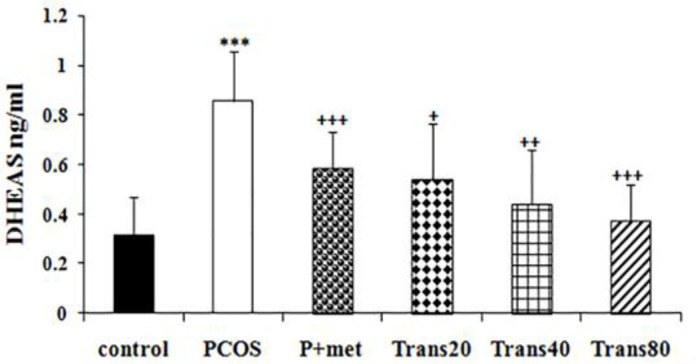
Effect of metformin and trans-anethole on DHEAS levels in control, PCOS, PCOS+metformin (P+met), and PCOS+trans-anethole (Trans 20, 40, and 80) groups. Values are expressed as mean±SEM. ***p<0.001, compared to the control group; ^+^p<0.05, ^++^p<0.01, and ^+++^p<0.001, compared to the PCOS group

There was a significant increase in serum DHEAS levels of the PCOS group compared to the control group (p<0.001). All trans-anethole-treated groups showed a significant decrease in DHEAS level compared to the PCOS group (p<0.05- p<0.001). Reduction of serum DHEAS level in the Trans80 group was more than other treated groups. No significant differences were found between DHEAS levels of the P+met group and trans-anethole-treated groups. DHEAS levels in groups treated with trans-anethole were not significantly different with the control group (Figure 2).

**The effect of metformin and trans-anethole on serum insulin level**

**Figure 3:**
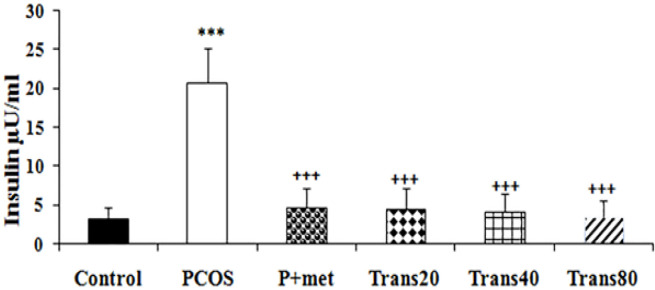
Effect of metformin and trans-anethole on insulin levels in the control, PCOS, PCOS+ metformin (P+met), and PCOS+trans-anethole (Trans 20, 40, and 80) groups. Values are expressed as mean±SEM. ***p<0.001, compared to the control group; ^+++^p<0.001, compared to the PCOS group

There was a significant increase in serum insulin levels of the PCOS group compared to the control group (p<0.001). Insulin levels were decreased significantly in the P+ met and all groups treated with trans-anethole compared to the PCOS group (p<0.001 for all). Insulin levels in groups treated with trans-anethole and metformin were not significantly different with control group (Figure 3).

**Effect of metformin and trans-anethole on serum cholesterol level**

**Figure 4:**
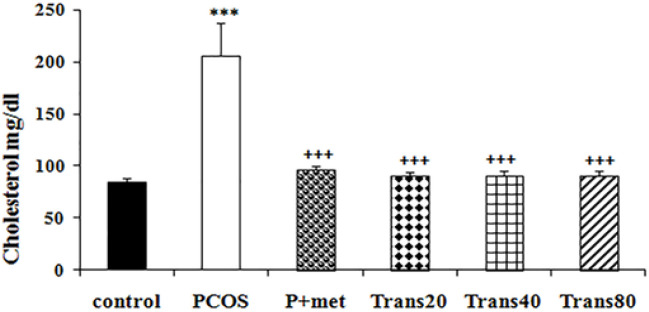
Effect of metformin and trans-anethole on cholesterol levels in the control, PCOS, PCOS+ metformin (P+met), and PCOS+trans-anethole (Trans 20, 40, and 80) groups. Values are expressed as mean±SEM. ***p<0.001, compared to the control group; ^+++^p<0.001, compared to the PCOS group

There was a significant increase in serum cholesterol levels of the PCOS group compared to the control group (p<0.001). Cholesterol levels were decreased significantly in the P+ met and all groups treated with trans-anethole compared to the PCOS group) p<0.001, for all groups (. The 

serum cholesterol levels of groups treated with trans-anethole and metformin were not significantly different from the control group (Figure 4).

**Effect of metformin and trans-anethole on serum triglyceride level**

**Figure 5:**
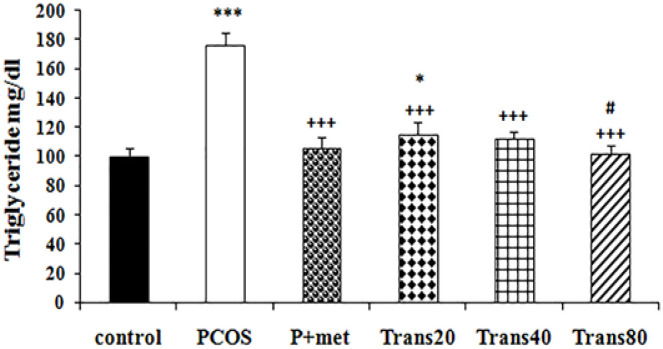
Effect of metformin and trans-anethole on triglyceride levels in the control, PCOS, PCOS+metformin (P+met), and PCOS+trans-anethole (Trans20, 40, and 80) groups. Values are expressed as mean±SEM. *p<0.05, and ***p<0.001, compared to the control group; ^+++^p<0.001, compared to the PCOS group; #p<0.05, comparison between the Trans20 and Trans80 group

There was a significant increase in serum triglyceride levels of PCOS group when compared to the control group (p<0.001). Groups treated with Trans80 and P+met showed a significant decrease in triglyceride levels compared to the PCOS group (p<0.001 for both groups). Moreover, the triglyceride level of Trans80 group was significantly (p<0.05) lower than the Trans20 group. Triglyceride level in groups treated with P+met and Trans80 were not significantly different from the control group (Figure 5). 

**Effect of metformin and trans-anethole on weight of PCOS rats**

**Figure 6:**
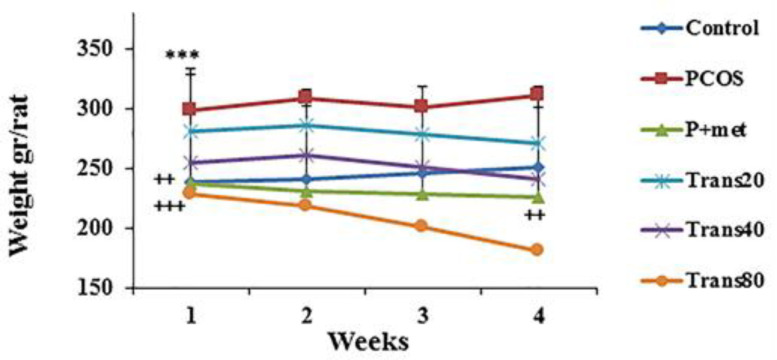
Effect of metformin and trans-anethole on weight in the control (weekly measurement was done from the onset of treatments), PCOS, PCOS+ metformin (P+met), and PCOS+trans-anethole (Trans 20, 40, and 80) groups. ***p<0.001, compared to the control group; ^+++^p<0.001, and ^++^p<0.01, compared to the PCOS group

There was a significant increase in weight gain of the PCOS group as compared to the control group (p<0.001). Weight gain in the groups Trans40, Trans80 and P+met was significantly lower than the PCOS group (p<0.01-p<0.001) (Figure 6).

**Effect of metformin and trans-anethole on water and food consumption**

**Figure 7:**
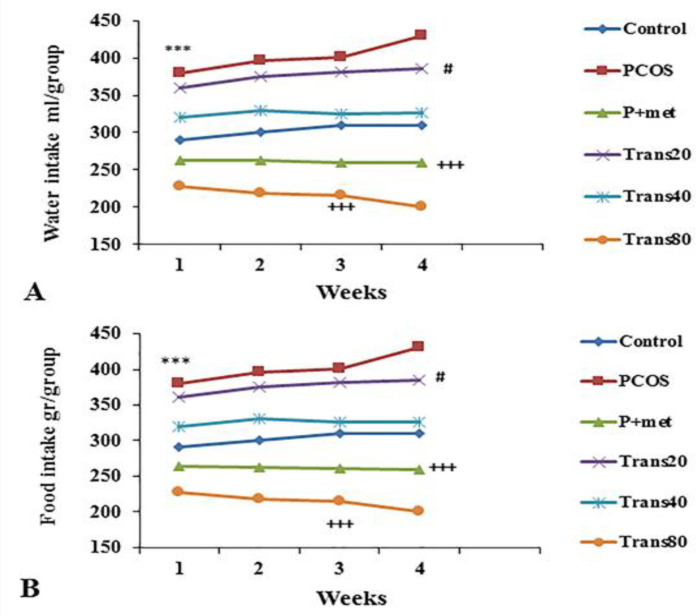
Effect of metformin and trans-anethole on water and food consumption in the control (weekly measurement was done from the onset of treatments), PCOS, PCOS+ metformin (P+met), and PCOS+trans-anethole (Trans20, 40, and 80) groups. ***p<0.001, compared to the control group; ^+++^p<0.001, compared to the PCOS group; #p<0.05 comparison between the Trans20 and Trans80 group

There was a significant increase in water and food consumption of the PCOS compared to the control group (p<0.001). Water and food consumption in the groups Trans80 and P+met significantly decreased compared to the PCOS group (p<0.001 and p<0.01, respectively). In addition, water and food consumption of the Trans80 group was significantly (p<0.05) lower than the Trans20 group (Figure 7).

## Discussion

PCOS patients have several hormonal and metabolic disorders, including high serum testosterone level, hyperlipidemia, hyperglycemia, insulin resistance, acne, hirsutism, and weight gain, and ovarian cysts, irregular menstruation, anovulation, infertility, high blood pressure, abdominal obesity, and hypercholesterolemia are also present in PCOS woman. In this study, we used testosterone injection to induce a reliable PCOS model (Connolly et al., 2015 ▶). PCOS induction resulted in a significant increase in testosterone, DHEAS, triglyceride and cholesterol levels and an increase in weight of rats compared to the control animals. Moreover, in the present study the above mentioned hormonal and metabolic changes led to an elevation of serum insulin and blood glucose in PCOS animals as was approved previously (Ilhan et al., 2020 ▶). These changes 

might be considered a response to insulin resistance and overproduction of insulin by pancreas due to insulin resistance (Baillargeon and Carpentier, 2007 ▶). It was shown that in such condition, the liver converts excess sugar into fat, leading to increased fat levels, and excess fat can increase the hormones levels when there is an increase in androgen level (Benson et al., 2008 ▶; Lerchbaum et al., 2012 ▶). Guo et al. (2019) ▶ study results are also in the line with our study. PCOS is associated with weight gain that disrupts ovulation and causes an increase in cholesterol and triglycerides. Factors such as an increase in the number of follicles (Guo et al., 2019 ▶) and increase in serum testosterone and DHEAS, blood lipids and sugar levels, and weight gain have been reported to be confirmatory indicators of establishing a PCOS model (O’Reilly et al., 2017 ▶). 

Currently, many drugs are used to treat PCOS, but none of them exerts a complete therapeutic effect and they have several side effects (Lowenstein, 2006 ▶). Recently, many investigations evaluated the efficacy of herbal medicines in the treatment of PCOS (Zehra and Khursheed, 2018 ▶). 

*F. vulgare *has estrogenic, anti-inflammatory and antioxidant effects; therefore, it is used to treat infertile women (Ghazanfarpour et al., 2018 ▶; Bayrami et al., 2020 ▶). These therapeutic effects of *F*. *vulgare* have been attributed to the active constituent of the plant, trans-anethole (Rather et al., 2016 ▶). *F. vulgare *extract decreased LH and testosterone levels in PCOS animals (Sadrefozalayi and Farokhi, 2014 ▶). 

In the present study, treatment of PCOS rats with trans-anethole 80 mg/kg significantly decreased testosterone and DHEAS against the PCOS group and returned the level of both androgens to near normal level by 30 days. 

Overproduction of IL6 in PCOS was shown to be an important factor in pathological changes, the regulation of steroids, the maturation of follicles, the processes of ovulation and sex hormone production from the ovaries (Roozenburg et al., 1997 ▶). Trans-anethole significantly inhibits the expression of interleukin (IL)-1b, IL-6, IL-10, and inducible nitric oxide synthase (Zuo et al., 2017 ▶), and thus inhibits inflammatory conditions. As a result, it reduces the production of follicle-stimulating hormone (FSH) and LH. Low levels of FSH and LH may lead to decreased follicle production and diminished serum levels of sex hormones (Parandin and Yousofvand, 2019 ▶). It was indicated that trans-anethole estrogenic properties can affect inflammation and improve ovulation processes, thus, it was recommended for treatment of primary  dysmenorrhea (Pellow and Scott, 2018 ▶) . Moreover, it was shown that *F*. *vulgare* extract can increase serum concentrations of FSH and decrease LH and testosterone in PCOS rats (Karampoor et al., 2014 ▶). In addition, treatment of PCOS animals with *F*. *vulgare* significantly regulated the levels of insulin, LH, fasting blood sugar, TG, TC, and testosterone (Bayrami et al., 2020 ▶). These results are in line with our findings about trans-anethole. In the present study, all PCOS rats treated with trans-anethole and metformin showed a decrease in serum level of insulin which was much closed to control animals. The study conducted by Sheikh et al. (2015) ▶, also supports our results; they showed that treatment with trans-anethole, decreased plasma insulin significantly, while the plasma glucose level remained elevated in PCOS in rats (Sheikh et al., 2015 ▶). Based on the results obtained from this study, it might be concluded that the elevated levels of insulin might have improved glucose utilization by peripheral tissues through promoting glucose uptake and metabolism, or accelerating the inhibition of hepatic gluconeogenesis which resulted in reduction of blood glucose levels (Ramachandran and Saravanan, 2013 ▶). 

The present study has showed a significant increase in insulin and testosterone, which indicates an association between a hyper androgenic and hyperinsulinemic status in PCOS (Allemand et al., 2009 ▶; Zeng et al., 2020 ▶). In groups treated with trans-anethole and metformin, the levels of cholesterol, and triglyceride were significantly reduced and reached near normal levels by 30 days. It was shown that *F*. *vulgare* extract reduced plasma cholesterol and triglyceride in PCOS mice (Al-Aridhi, 2014 ▶). Previous studies have shown that fennel might shift liver total lipid value to normal level; action of fennel on reducing lipids could be attributed to its anti-oxidative properties and radical scavenging activity as *F*. *vulgare* extract could significantly increase HDL level (Radwan et al., 2019 ▶). Furthermore, it has been indicated that a herbal formulation products from *F*. *vulgare*, could delay upper gastrointestinal transit which promotes a decrease in fat and sugar absorption (Sheikh et al., 2015 ▶). 

In our study, the weight of animals and the amount of water and food intake in the PCOS group showed a significant increase when compared to the control group; trans-anethole 40 and 80 mg/kg, and metformin showed the greatest effect on weight loss in PCOS rats. Findings of Ribstein et al. (1999) ▶ demonstrated that the body weight of PCOS rats treated with fennel herb was significantly decreased, which might be due to trans-anethole inhibitory effect on trypsin, reduction of food intake, and increase in satiety (Ribstein et al., 1999 ▶). Findings of the present study showed that trans-anethole 80 mg/kg has the greatest effect up on many factors in PCOS animals; therefore, it might be concluded that the effect of trans-anethole on PCOS is dose-dependent. 

Metformin could induce ovulation, improved the menstrual cycle (Nestler and Jakubowicz, 1997 ▶), and it is used in treatment of DMT2 in PCOS woman (Garimella et al., 2016 ▶). In this study, the anti-androgenic effect of trans-anethole was comparable to metformin as standard drug.

These results indicated that trans-anethole especially at the dose of 80mg/kg has a beneficial effect on PCOS complications by ameliorating PCOS biochemical and hormonal changes. Therefore, trans-anethole might be suggested as a beneficial remedy for PCOS patients after approving in future clinical studies.
